# Real-world use of polidocanol foam sclerotherapy for hemorrhoidal disease: insights from an international survey and systematic review with clinical practice recommendations

**DOI:** 10.1007/s13304-025-02258-2

**Published:** 2025-06-06

**Authors:** Gaetano Gallo, Ugo Grossi, Veronica De Simone, Arcangelo Picciariello, Elia Diaco, Pin Fan, Hongbo He, Jun Li, Hongcheng Lin, Marco La Torre, Rita Laforgia, Pierluigi Lobascio, Hui Ma, Francesco Pata, Roberto Perinotti, Vincent De Parades, Mauro Pozzo, Alberto Realis Luc, Paulo Salgueiro, Adam Skowronski, Pingliang Sun, Mario Trompetto, Roberta Tutino, Chen Wang, Zhenyi Wang, Zhenquan Wang, Jiong Wu, Yuru Zhang, Shipeng Zhao, Xiandong Zeng, Vitor Fernandes, Karl-Heinz Moser, Donglin Ren, Pierpaolo Sileri, Gianpiero Gravante

**Affiliations:** 1 Department of Surgery, Sapienza University of Rome, Rome, Italy; 2 Surgery Unit 2, Regional Hospital Treviso ‘Cittadella della Salute’, Piazzale Ospedale 1, 31100 Treviso, Italy; 3 Proctology and Pelvic Floor Surgery Unit, Ospedale Isola Tiberina-Gemelli Isola, 00186 Rome, Italy; 4 Department of Experimental Medicine, University of Salento, Lecce, Italy; 5 Minerva Surgical Service, Catanzaro, Italy; 6 The First Affiliated Hospital of USTC, Anhui Provincial Hospital, Hefei, China; 7 West China Hospital of Sichuan University, Chengdu, China; 8 The Affiliated Hospital of Southwest Medical University, Luzhou, China; 9 The Sixth Affiliated Hospital of Sun Yat-Sen University, Guangzhou, China; 10 Department of Surgery, Ospedale Cristo Re, Rome, Italy; 11 Department of Precision and Regenerative Medicine and Jonic Area (DiMePRe-J), Section of Surgery, General Surgery Unit—Hospital University of Bari, Piazza Giulio Cesare 11, 70124 Bari, SE Italy; 12 The First Affiliated Hospital of Guangxi Medical University, Nanning, China; 13 Department of Pharmacy, Health and Nutritional Sciences, University of Calabria, Cosenza, Italy; 14 General Surgery, SS Colo-Rectal and Proctological Surgery, Biella Hospital, Ponderano, Biella, Italy; 15 Institut Léopold Bellan, Groupe Hospitalier Paris Saint-Joseph, Service de Proctologie Médico-Chirurgicale, Paris, France; 16 Department of Colorectal Surgery, S. Rita Clinic, Vercelli, Italy; 17 Department of Gastroenterology, Hospital de Santo António, Centro Hospitalar Universitário Do Porto, Largo Prof. Abel Salazar, 4099-001 Porto, Portugal; 18 Centrum Medyczne PZU Zdrowie Artimed, Kielce, Poland; 19 Department of General and Emergency Surgery, AOU Città della Salute e della Scienza, Turin, Italy; 20 Longhua Hospital, Shanghai University of Traditional Chinese Medicine, Shanghai, China; 21 Yueyang Hospital of Integrated Traditional Chinese and Western Medicine, Shanghai University of Traditional Chinese Medicine, Shanghai, China; 22 The Second Affiliated Hospital of Hunan University of Traditional Chinese Medicine, Hunan University of Chinese Medicine, Changsha, China; 23 Beijing Rectum Hospital, Beijing, China; 24 Hebei Medical University Third Hospital, Shijiazhuang, Hebei China; 25 the Fourth People’s Hospital of Shenyang, Shenyang, Liaoning China; 26 Gastroenterology Department, Hospital Garcia de Orta, Avenida Torrado da Silva, 2801-951 Almada, Portugal; 27 “Suedstadt” Surgical Group Practise, Karolingerring 31, 50678 Cologne, Germany; 28 Colorectal Surgery Unit, IRCCS San Raffaele Scientific Institute, Vita-Salute University, Via Olgettina 60, 20132 Milan, Italy; 29 Department of General Surgery, Azienda Sanitaria Locale ASL Lecce, Casarano, Italy

**Keywords:** Polidocanol foam, Expert survey, Hemorrhoidal disease, Sclerotherapy, Systematic review

## Abstract

Polidocanol foam sclerotherapy has gained increasing attention as a minimally invasive treatment for hemorrhoidal disease (HD). However, significant variability exists in its clinical application regarding patient selection, procedural techniques, and postoperative management. This study aimed to assess real-world practice patterns among international experts, summarize existing evidence through a systematic literature review, and develop evidence-based clinical practice recommendations. A systematic review was conducted in MEDLINE, EMBASE, and CENTRAL to identify studies evaluating polidocanol foam sclerotherapy for HD. A total of 20 studies met the inclusion criteria. Additionally, an international survey was distributed to 30 experts in proctology and colorectal surgery to explore variations in indications, perioperative management, technique, and follow-up. Survey responses were analyzed descriptively to identify common trends and areas of divergence. Subsequently, based on both the experts’ opinions and the results of the survey, a Delphi method was employed to produce clinical practice recommendations. The questions for the Delphi process were developed by the authors leading the project, followed by a detailed discussion with the whole panel of experts. Most experts (90%) reported using polidocanol foam sclerotherapy primarily for Goligher grade II HD, with 67% extending its use to grade III cases. Preoperative bowel preparation and anesthesia use varied widely. The preferred concentration was 3% polidocanol, with 2 mL injected per hemorrhoidal pile in most cases. Post-procedural care lacked standardization, though stool softeners and flavonoids were commonly recommended. Compared to rubber band ligation, polidocanol foam was perceived as having higher success rates (88.3% vs. 66.7%) and lower recurrence rates (16.1% vs. 41.2%). Adverse events were infrequent. The present article offers a comprehensive suite of clinical practice expert-based recommendations concerning the use of polidocanol foam sclerotherapy for HD. However, there is still significant variation in its application. These findings highlight the need for standardized guidelines and further research to optimize procedural strategies and long-term outcomes.

**Supplementary Information:**

The online version contains supplementary material available at 10.1007/s13304-025-02258-2.

## Introduction

Hemorrhoidal disease (HD) affects approximately 4% of the population [1]. Its management is typically guided by the Goligher classification and the impact of symptoms on the patient’s quality of life [1, 2]. While grade IV HD is usually treated with surgical excision, lower-grade cases can often be managed with less invasive techniques, including office-based procedures such as sclerotherapy and rubber band ligation (RBL), when conservative treatments fail [3].

Sclerotherapy dates back to the nineteenth century as the first minimally invasive treatment described for HD [4]. Despite being easy to perform and initially showing promising efficacy, concerns over the safety of early sclerosing agents relegated this technique to a second-line treatment after RBL [3, 5]. This preference was reaffirmed in 2018 by a large survey involving more than 32,000 patients, which reported that over 90% of grade II HD were still treated with RBL [6]. More recently, polidocanol has emerged as a sclerosing agent, first gaining widespread use in the treatment of varicose veins [7], before being successfully applied to HD. Its improved safety profile progressively replaced phenol oil as the preferred sclerosing agent [8, 9], and the introduction of polidocanol foam further enhanced its efficacy compared to the original liquid formulation [10]. These advancements have led to a shifting paradigm, with sclerotherapy increasingly being considered a first-line treatment for symptomatic grade II–III HD, potentially reserving RBL for recurrent cases [11, 12].

Despite the growing interest in polidocanol foam sclerotherapy, there is significant variability in how the procedure is performed in clinical practice, including aspects such as patient selection, technique, dosing, and follow-up. This study aims to describe the real-world practice patterns of international experts regarding polidocanol foam sclerotherapy for HD, providing an overview of its current application and highlighting variations in procedural strategies. In addition, a systematic review of the literature was conducted to summarize the available evidence on polidocanol foam sclerotherapy and provide a contextual foundation for the survey-based study. Clinical practice recommendations were formulated based on both the experts’ opinions and the results of the review.

## Materials and methods

### Systematic literature review

**Fig. 1:**
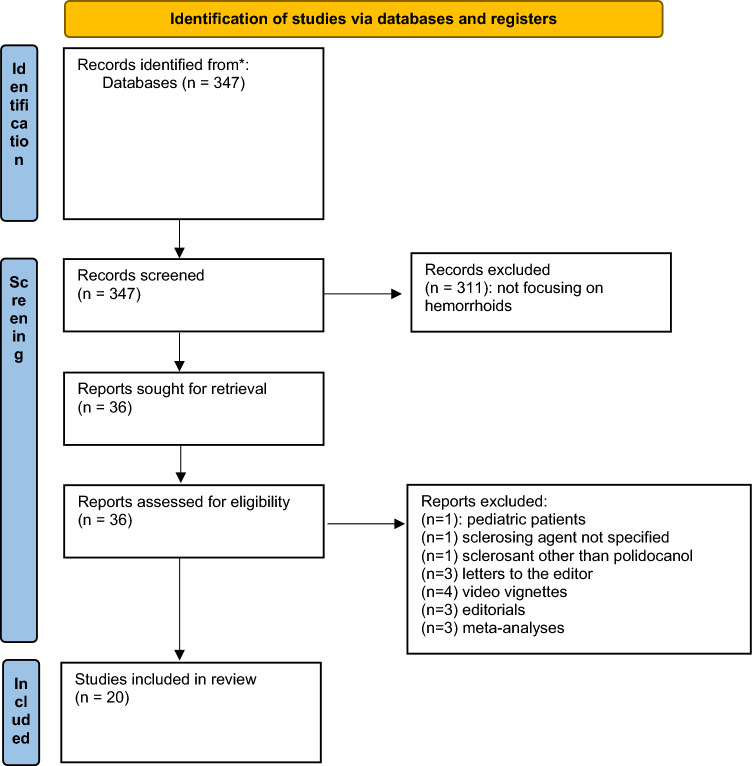
PRISMA diagram

Given the variability in clinical practice and the absence of standardized guidelines for polidocanol foam sclerotherapy, a systematic review of the literature was conducted to provide an evidence-based background on its indications, techniques, and outcomes. The authors developed the protocol for review, detailing pre-specified methods of analysis and eligibility of the studies, in line with the 2020 Preferred Reporting Items for Systematic Reviews and Meta-analyses (PRISMA) guidance (Fig. 1) [13].

A structured search strategy was employed using two sets of keywords: one related to HD and one specific to polidocanol foam sclerotherapy. The keywords used for HD were “hemorr”* and “haemorrh”* (truncated terms), while those for polidocanol foam sclerotherapy included “polidocanol”, “foam”, and “sclerotherapy”. The search was performed in MEDLINE, EMBASE, and Cochrane Central Register of Controlled Trials (CENTRAL) until January 2025. Additionally, the Current Controlled Trials database (www.controlled-trials.com) was searched for ongoing randomized trials.

Studies were considered eligible if they investigated the use of polidocanol sclerotherapy for HD and provided original data on newly treated patients. This included cohort studies, case–control studies, and randomized controlled trials. Conversely, studies were excluded if they focused on pediatric patients, involved sclerosants other than polidocanol or failed to specify the product used. Additionally, letters to the editor, expert opinions, editorials, clinical practice guidelines, and video vignettes were not considered. Although meta-analyses and systematic reviews were excluded from direct inclusion, their reference lists were carefully examined to identify further relevant studies. Two independent reviewers (GGa and GGr) conducted a systematic screening of titles and abstracts, followed by full-text assessment of eligible studies. Discrepancies were resolved by discussion or consultation with a third author (UG). The findings of the systematic review were used to contextualize the results of the survey study.

### Expert survey study

#### Panel composition and survey development

To explore current practice patterns in polidocanol foam sclerotherapy, a structured survey was developed and distributed to an international panel (*International Sclerotherapy Group, ISG*) consisting of experts in proctology, colorectal surgery, and minimally invasive treatments for HD (Appendix 1). Experts from multiple geographic regions were selected based on a combination of documented clinical and scientific experience in the management of HD. While all had a particular interest in polidocanol foam sclerotherapy, they were also proficient in performing the full range of therapeutic procedures necessary to achieve a tailored approach to HD. All identified surgeons were previously invited by email to attend an introductory video conference outlining the principles of the project.

The questionnaire was designed, in accordance with the Consolidated criteria for Reporting Qualitative Research (COREQ) and the Checklist for Reporting Results of Internet E-Surveys (the CHERRIES statement) [14], by 5 authors leading the project (GGa, UG, VDS, AP, & GGr), using LimeSurvey GmbH®, to assess real-world variations in clinical practice, including indications, perioperative management, technical aspects of the procedure, and post-treatment follow-up. The survey covered the following key domains related to procedural decision-making: patient selection and preoperative preparation, injection technique (volume, concentration, anatomical sites), post-procedural management and follow-up, efficacy outcomes and patient-reported satisfaction, safety considerations and complications. The questionnaire was iteratively refined based on expert feedback before distribution. It was then electronically administered to the group.

#### Survey administration and data collection

The survey was conducted online, with participants responding anonymously to ensure unbiased reporting of their clinical practice. Responses were collected over a specified period, and data were analyzed descriptively to identify common trends and areas of variation. The survey aimed to document variations in practice without the goal of achieving expert consensus or producing formal guidelines. Subsequently, based on both the experts’ opinion as well as on the results of the search a Delphi method, with questions produced by the authors leading the project, was used to produce clinical practice recommendations after a detailed discussion. The leading group established 75% agreement as necessary to reach a consensus [15].

Considering the scarcity of high-quality publications, the Delphi method was selected as the optimal approach to mitigate bias during the recommendation formulation process. A maximum of two voting rounds was defined a priori. Discordances amongst experts were resolved by open discussions.

## Results

### Systematic literature review

A total of 20 studies met the inclusion criteria and were analyzed in detail [8, 16–34]. Several studies were excluded based on pre-specified criteria: one study was excluded due to its focus on pediatric patients [35], one did not specify the sclerosing agent used [36], and one study used a sclerosant other than polidocanol [37]. Additionally, three studies were excluded as letters to the editor [38–40], four were video vignettes [41–44], and three were editorials [12, 45, 46]. Three meta-analyses [47–49] were reviewed to extract additional studies that were not identified in the initial search (Fig. 1).

Nearly half of the studies (9/20, 45%) were conducted in Italy [16, 22–25, 28, 30–32]. The majority were prospective, including six randomized controlled trials [8, 18, 21, 26, 33, 34]. Only three studies were retrospective [22, 29, 32], while eight studies were multicenter investigations [8, 23, 25, 27–30, 34] (Table 1). Table 1 summarizes the main characteristics of the studies, including clinical indications for polidocanol foam sclerotherapy. Most studies targeted patients with grade I–III hemorrhoidal disease, with particular consideration to cases involving bleeding diathesis, patients unfit for surgery, or those with recurrence after previous treatments such as RBL. The number of treatment sessions ranged from one to three, and clinical success rates varied between 68 and 100%, with low recurrence rates reported at a median follow-up of 12 months. Reported complications were generally mild and self-limiting. Table 2 provides an overview of adverse events, with pain, pruritus, and transient bleeding being the most frequently observed. Serious complications were rare.

**Table 1:** Summary of studies on polidocanol foam sclerotherapy for hemorrhoidal disease

Authors	Year	Country	Type of trial	Number of centers involved	Polidocanol	%	Total patients	Goligher grade	Number of sessions^a^	f/up	Success rate
Moser et al. [8]	2013	Germany	Prospective randomized	Multicenter	Foam vs. liquid	3%	66 vs. 64	I	1–3	3 months	88% vs. 69%
Ronconi et al. [16]	2018	Italy	Prospective	Single center	Foam (endoscopic)	3%	615	I–IV	1–5	1 year	86% (prolapse reduction)
Fernandes et al. [17]	2019	Portugal	Prospective	Single center	Foam	2%	2000	II–IV	1–4	4 months	86%
Shen et al. [18]	2019	China	Prospectiverandomized	–	Foam vs. liquid	–	55 vs. 53	–	–	–	87.3% vs. 69.8%
Makanjuola et al. [19]	2020	Nigeria	Prospective	Single center	Foam vs. RBL	3%	37 vs. 37	I–III	1–3	3 months	No difference
Shekhar et al. [20]	2020	India	Prospective	Single center	Foam	3%	50	I–III	1–3	3 months	96%
Qi et al. [21]	2020	China	Prospective randomized	Single center	Foam vs. conservative treatment	–	120 vs. 100	–	–	3 months	Improvement of various scores
Lobascio et al. [23]	2021	Italy	Prospective	Multicenter	Foam	3%	66	II–III	1–2	1 year	86%
Lisi et al. [24]	2021	Italy	Prospective	Single center	Foam	3%	10	III–IV	1	1 month	100%
Goglia et al. [25]	2022	Italy	Prospective	Multicenter	Foam	3%	50	II	1–3	3 months	91.7%
Salgueiro et al. [26]	2022	Portugal	Prospective randomized	Single center	Foam vs. RBL	3%	60 vs. 60	I–III	1–3	1 year	88% vs. 67%
Salgueiro et al. [27]	2022	Portugal	Prospective	Multicenter	Foam	3%	228	I–III	1–3	1 year	93.4%
Pata et al. [22]	2022	Italy	Retrospective	Single center	Foam + RBL (Sclerobanding)	3%	125	II–III	–	1 year	–
Gallo et al. [28]^b^	2022	Italy	Prospective	Multicenter	Foam	3%	183	II	1–3	1 year	95.6%
Figuereido et al. [29]	2022	Portugal	Retrospective	Multicenter	Foam	2%	243	I–III	–	1 year (mean)	90.1%
Gallo et al. [30]^b^	2023	Italy	Prospective	Multicenter	Foam	3%	183	II	1–3	3 years	90.2%
Pata et al. [31]	2023	Italy	Prospective	Single center	Foam + RBL (Sclerobanding)	3%	51	II–III	–	2 years (mean)	96%
Lobascio et al. [32]	2023	Italy	Retrospective	Single center	Foam vs. Mucopexy/Deharterialization	3%	150 vs. 109	II–III	1–2	2 years (mean)	93.3% vs. 93.5%
Neves et al. [33]	2023	Portugal	Prospective randomized	Single center	Foam vs. Mucopexy/Deharterialization	3%	24 vs. 22	II–III	1–2	2 months	91.7% vs. 68.2%
Qu et al. [34]	2024	China	Prospective randomized	Multicenter	Foam (endoscopic) + RBL (Sclerobanding) vs. RBL	3%	98 vs. 97	II–III	–	1 year	88.8% (no prolapse recurrence)

^a^Most patients achieved complete resolution of their symptoms after one session of treatment, only few needed a second or even third session

^b^Two articles involved the same cohort of patients followed at different follow-ups (one and three years, respectively)

*f/up* follow-up, *RBL* rubber band ligation

**Table 2:** Postoperative complications following polidocanol foam sclerotherapy for hemorrhoidal disease

Complications	N patients/total	%	95%CI _(Lower; Upper)_
Postoperative pain (moderate or severe)	162/2024	8.0%	(6.8; 9.2)
Postoperative pain (mild)	63/1029	6.1%	(4.7; 7.6)
Itching	29/980	2.9%	(1.9; 4.0)
Postoperative bleeding	11/389	2.8%	(1.2; 4.5)
Tenesmus	10/365	2.7%	(1.0; 4.4)
Soiling	6/365	1.6%	(0.3; 2.9)
External thrombosis	13/2365	0.5%	(0.3; 0.9)
Prostatitis	1/365	0.3%	(0.0; 0.8)
Significant bleeding^a^	4/2024	0.2%	(0.0; 0.4)
Rectal abscess	2/2000	0.1%	(0.0; 0.2)

Rates were pooled from multiple studies included in the systematic review

^a^Two patients were on double antiaggregant/anticoagulant [17]

### Expert survey study

A total of 30 experts in proctology, colorectal surgery, and minimally invasive treatments for HD participated and completed the survey. The majority were male (25/30, 83.3%), with most participants aged between 40 and 60 years (24/30, 80%). Geographically, the highest number of surgeons came from China (14/30, 46.7%), followed by Italy (11/30, 36.7%), Portugal (2/30, 6.7%) and France, Germany and Poland (1/30, 3.3%), and the majority worked in teaching hospitals (24/30, 80%). Over half were colorectal surgeons (16/30, 53.3%), 5 anorectal surgeons/proctologists (16.7%), 4 general surgeons (13.3%), 4 gastroenterologists (13.3%) and 1 angiologist (3.3%) (Appendix 1).

The survey documented how polidocanol foam sclerotherapy is currently performed in clinical practice. Responses were grouped into five key areas: (1) indications and contraindications, (2) perioperative management, (3) procedure details, (4) postoperative care and follow-up, (5) comparative effectiveness and safety profile.

#### Indications and contraindications

Most respondents (27/30, 90%) reported using polidocanol foam sclerotherapy primarily for Goligher grade II HD. The procedure was particularly favored in elderly patients with significant comorbidities (23/30, 76.7%). Additionally, 16/30 (67%) of respondents considered it suitable for grade III HD, though its use in grade IV cases was less common (8/30, 26.7%). A relevant subset of experts reported using sclerotherapy in patients on anticoagulation therapy (18/30, 60%). The “bridge to surgery” option, widely adopted during the COVID-19 pandemic, was considered in 13 out of 30 respondents (43.3%). In rare cases, polidocanol foam was employed in patients with inflammatory bowel disease (IBD) (2/30, 6.6%), pregnancy (4/30, 13.3%) or as part of a combined approach with RBL (11/30, 36.7%) or excisional procedures (1/30, 3.3%). Regarding contraindications, the most commonly reported were anal abscess (22/30, 73.3%) and anal stenosis (17/30, 56.7%).

*The ISG recommends the use of Sclerotherapy for patients with grade I-III HD (Strong Consensus: 100%). Moreover, the procedure could be performed in elderly patients, in patients on anticoagulation therapy, in medically-controlled-IBD and in combination with excisional hemorrhoidectomy (Strong Consensus: 93.3%). The application of sclerotherapy for patients with grade IV HD must be aimed exclusively at treating symptoms, even with the intent of bridging to surgery (Strong Consensus: 83.3%).*

#### Perioperative management

Preoperative preparation for polidocanol foam sclerotherapy was not standardized, with notable variations in clinical practice among surgeons. The majority of practitioners incorporated some form of medical management before the procedure, with stool softeners being the most commonly prescribed (15/30, 50%), followed by high-fiber diets (12/30, 40%) and systemic flavonoids (11/30, 36.7%). A smaller proportion (4/30, 13%) recommended topical flavonoids. The duration of pre-treatment also varied, with most surgeons opting for a four-week course (6/30, 31.6%), while others preferred a shorter, two-week regimen (3/30, 15.8%).

The setting in which the procedure was performed also differed among respondents. Just over half of the surgeons (16/30, 53.3%) conducted sclerotherapy in an outpatient office setting, while 9/30 (30%) preferred a day surgery unit, and 5/30 (16.7%) performed the procedure in an inpatient setting.

*Anesthesia use was another area of variability. Half of the surgeons (15/30, 50%) performed the procedure without any anesthesia, whereas nearly a third (9/30, 30%) used lidocaine gel for local analgesia. Preoperative bowel preparation was also inconsistent: enemas (single or double the night before and a few hours before the procedure) were administered in 22/30 cases (56.7% single; 16.7% double), whereas 7 surgeons (50%) opted to proceed without any bowel preparation.*

*The ISG recommends the use of a 4-week preoperative therapy* *with local and systemic flavonoids, stool softeners, high-fibers diet to strengthen the effectiveness of sclerotherapy* *(Strong Consensus: 100%)*. *A preoperative bowel preparation with single or double enemas could be chosen (Strong Consensus: 93.3%). The procedure must be performed in an outpatient setting if possible and if the local organization allows it using lidocaine gel (Strong Consensus: 100%).*

#### Procedure details

The preferred concentration of polidocanol foam varied among surgeons, with the majority (17/30, 65.4%) favoring a 3% solution. A smaller proportion opted for a lower concentration, with 1% being used in 7/30 cases (26.9%) and 2% in 2/30 cases (7.7%). Foam preparation methods were also diverse, with nearly half of the surgeons (12/30, 46.1%) using the Tessari method, while 10/30 (38.5%) and 2/30 (7.7%) relied on the EasyFoam kit© and automated devices (i.e. Varixio ©), respectively, reflecting the recent evolution of new preparation methods.

Variability was also observed in the choice of injection needle gauge. The most frequently used diameters were 20G (7/30, 23.3%), followed by 22G (5/30, 16.7%) and 23G (4/30, 13.3%). Regarding the injection technique, the majority of surgeons (19/30, 63.3%) preferred an intra-pile approach, while a smaller proportion (10/30, 33%) opted for submucosal injection.

The volume of foam injected per hemorrhoidal pile was typically 2 mL (20/30, 66.7%), though in some cases it was increased to 3 mL (5/30, 16.7%). The total volume administered per session varied, ranging between 6 and 14 mL (26/30, 86.7%), with most surgeons (25/30, 83.3%) adjusting the dose based on hemorrhoidal grade.

*The ISG recommends the use of 3% liquid polidocanol to generate the foam, if possible, with an automated, non-operator dependent method, and the injection should be performed intra-pile with an open-ended anoscope and a 20 G needle and with a tailored use of 2–4 cc of foam per pile based on the degree of HD to be treated* *(Strong Consensus: 83.3%).*

#### Postoperative care and follow-up

Post-treatment care varied among surgeons, with most recommending supportive measures to enhance recovery and symptom resolution. Stool softeners were the most frequently prescribed intervention (23/30, 76.7%), followed by increased water intake (20/30, 66.7%) and systemic flavonoids (17/30, 56.7%).

Patients were typically reassessed within two to four weeks after the procedure, with follow-ups scheduled at two weeks in 10/30 cases (35.7%) and at four weeks in 9/30 cases (32.1%). Treatment success was primarily determined based on patient-reported outcomes (24/30, 80%), often supplemented by anoscopic evaluation (20/30, 66.7%) to assess the persistence of symptoms or resolution of hemorrhoidal disease.

A second session of sclerotherapy was generally not planned as part of routine care. However, in cases where patients continued to experience symptoms, additional sessions were considered on an individual basis (23/30, 76.7%).

*Postoperative therapy should be consistent with preoperative therapy and should be administered for at least 4 weeks after the procedure* *(Strong Consensus: 83.3%)*. *The ISG suggest a follow-up visit within 4 weeks of the injection to evaluate on an individual basis the execution of a possible further sclerotherapy injection* *(Strong Consensus: 100%).*

*A 4-week window must be respected between the first and second injection while subsequent procedures can be performed, on a symptomatic basis, after at least 3 months* *(Strong Consensus: 100%).*

#### Comparative effectiveness and safety profile

Survey responses indicated that polidocanol foam sclerotherapy is perceived as having superior outcomes compared with RBL and dearterialization procedures. Success rates were higher with polidocanol foam (88.3%) compared to RBL (66.7%), while recurrence rates were notably lower for sclerotherapy (16.1% vs. 41.2%) [26]. Additionally, complication rates were reduced in the sclerotherapy group (10.0%) compared to RBL (30.0%) [26]. Postoperative pain was also reported as lower among patients undergoing sclerotherapy compared to those treated with RBL [19, 50]. A meta-analysis comparing sclerotherapy to RBL confirmed these findings, supporting the safety and efficacy of polidocanol foam [48]. When compared to dearterialization and mucopexy, sclerotherapy demonstrated similar success rates but a more favorable safety profile, with fewer postoperative complications such as hemorrhage, thrombosis, and perineal abscess [32, 33]. Mucopexy was found to be associated with a sixfold higher risk of complications compared to sclerotherapy [33].

In terms of safety, adverse events were infrequent (Table 2). Mild pain was reported by 8.0% of patients, typically short-lived and self-limiting. Bleeding requiring hospital admission occurred in four cases, predominantly in patients receiving anticoagulant therapy. Additionally, two cases of rectal abscesses were documented, both requiring surgical drainage. Despite these rare complications, polidocanol foam was generally well-tolerated, reinforcing its safety profile in the treatment of HD.

*The ISG recommends the use of sclerotherapy as the first approach in patients with grade I-III HD. A combination with rubber band ligation may be considered in patients who are not at risk of postoperative bleeding* *(Strong Consensus: 100%).*

## Discussions

This is the first systematic review that focuses exclusively on the role of polidocanol foam by combining data from the literature and the experience of surgeons dedicated to the treatment of HD. The findings of the systematic review support the use of polidocanol foam sclerotherapy primarily in patients with grade I–III HD, and a particular interest application involves patients with bleeding diathesis, unfit for surgery, or with recurrent bleeding. These indications are consistent across the reviewed literature (Table 1). Moreover, the safety profile appears favorable, with a low incidence of significant complications (Table 2), reinforcing its role as a minimally invasive treatment option in selected patients.

### Indications and contraindications for polidocanol foam sclerotherapy

The findings from this expert survey describe the role of polidocanol foam sclerotherapy in the management of Goligher grade II HD, aligning with previous studies that demonstrated its efficacy in this subgroup [8, 20, 25]. In addition to its well-established role in grade II cases, polidocanol foam was also deemed appropriate for grade III hemorrhoids by a majority of experts (67%), particularly in elderly patients with significant comorbidities (76.7%). This finding is supported by literature indicating that polidocanol foam is well-tolerated in frail populations and offers symptom relief comparable to more invasive procedures, with fewer adverse events [26, 28, 30]. While the use of polidocanol foam for grade IV hemorrhoids remains limited (26.7%), some reports suggest its role as a bridge-to-surgery treatment, a strategy that gained prominence during the COVID-19 pandemic [24, 51]. Contraindications identified in the expert panel, including anal abscess (73.3%) and anal stenosis (56.7%), are consistent with previous reports highlighting the risks of tissue necrosis and impaired healing in these settings [27, 29, 31]. Patients with IBD represented a rare indication (3.3%), an area that remains controversial given concerns about mucosal healing and potential exacerbation of underlying pathology [56].

These findings reinforce the evolving role of polidocanol foam sclerotherapy as a primary treatment for grade II hemorrhoids, with promising indications for grade III cases, particularly in high-risk patients.

### Perioperative management

The findings of the survey highlight the lack of standardization in preoperative management, mirroring the heterogeneity observed in the literature. While stool softeners (50%) and high-fiber diets (40%) were commonly recommended, previous studies have not consistently demonstrated the necessity of routine bowel regulation prior to sclerotherapy [22, 31]. Flavonoids, commonly used in HD, could reduce symptoms and prepare tissues for the local injections [52, 53]. The variability in preoperative medical management underscores the need for future trials to evaluate its impact on outcomes.

A key point of divergence among experts was the setting of the procedure. The majority performed sclerotherapy in an outpatient setting (53.3%), whereas others preferred a day surgery unit (30%) or inpatient setting (16.7%). This discrepancy reflects differences in local healthcare infrastructure and physician preference rather than evidence-based practice, as most studies have shown comparable outcomes irrespective of setting [24, 28].

The use of anesthesia was also inconsistent, with 50% of surgeons forgoing anesthesia altogether, while 30% preferred lidocaine gel for local analgesia. Prior studies have suggested that polidocanol foam injection is generally well-tolerated without anesthesia, but further comparative trials could help determine if topical agents offer significant benefits in pain control [17, 22, 31].

### Procedure details

The survey revealed a strong consensus regarding the preferred polidocanol concentration, with 65.4% favoring a 3% solution. This aligns with published studies demonstrating superior clinical outcomes with this concentration compared to lower strengths, such as 1% and 2% [8, 20, 25]. The Tessari method was the most common technique for foam preparation (46.1%), in line with previous literature supporting its ability to produce a stable and homogenous microfoam with enhanced sclerosing capacity [17].

There was notable variation in injection techniques, with most surgeons preferring an intralesional approach (63.3%) over a submucosal injection (33%). Existing evidence supports both techniques but suggests that intralesional injection may enhance sclerosant contact with the vascular plexus, leading to improved outcomes [8, 17, 28]. Additionally, the survey findings confirmed that the volume per nodule was typically 2 mL (66.7%), while the maximum volume per session ranged between 6 and 14 mL (86.7%). These results align with prior recommendations advocating for dose adjustments based on hemorrhoidal grade to optimize efficacy and minimize complications [17, 20, 27].

### Postoperative care and follow-up

Post-treatment care varied among experts, with stool softeners (76.7%) and increased water intake (66.7%) being the most commonly prescribed interventions. The use of systemic flavonoids (56.7%) was also common, reflecting prior studies that suggest a potential role in reducing post-procedural symptoms, though robust evidence remains limited [16, 22, 25, 31].

Follow-up practices also showed variation, with reassessments occurring at two weeks (35.7%) or four weeks (32.1%). These intervals align with existing literature, which suggests that clinical response and the need for additional treatment should be evaluated within the first month post-procedure [24, 28]. Notably, success was primarily assessed using patient-reported outcomes (80%), a trend that aligns with recent research advocating for a shift toward patient-centered metrics over purely anatomical findings [18, 34].

### Comparative effectiveness and safety profile

Comparative studies consistently support the superiority of polidocanol foam over RBL in terms of success rates (88.3% vs. 66.7%) and lower recurrence (16.1% vs. 41.2%) [26]. Meta-analyses further reinforced these advantages, demonstrating that sclerotherapy is associated with fewer postoperative complications compared to dearterialization and mucopexy [32, 33]. Importantly, mucopexy was found to carry a sixfold higher risk of complications compared to sclerotherapy [33].

The safety profile of polidocanol foam was favorable, with low rates of significant adverse events. Mild pain was reported in 8.0% of patients, typically short-lived and self-limiting. Four cases of significant bleeding requiring hospitalization were observed, primarily among patients on anticoagulant therapy, aligning with previous reports highlighting the need for caution in this subgroup [17, 27]. Additionally, two cases of rectal abscesses required surgical drainage, an infrequent but recognized complication [17].

### Future directions

The increasing adoption of polidocanol foam as a first-line treatment for grade II–III HD is reflected in both expert survey responses and an expanding body of literature. However, several questions remain unanswered. The long-term durability of polidocanol foam relative to RBL and other techniques needs further study, as do optimal dosing strategies and injection techniques. Additionally, the role of combination therapies, such as sclerobanding, warrants further investigation, given preliminary data suggesting enhanced efficacy in certain patient populations [22, 31, 34, 39, 42, 45].

From a clinical perspective, the lack of standardization in preoperative and postoperative management highlights the need for guideline development and prospective studies. Finally, real-world data on patient-reported outcomes will be crucial in shaping future recommendations and optimizing treatment selection.

### Limitations

A number of limitations must be acknowledged. A key challenge in analyzing the available literature was the lack of a standardized definition of treatment success, a common issue in proctologic studies [54], as well as the failure to consider most symptoms in the Goligher classification [55]. Reported success rates varied depending on the criteria used, ranging from partial symptom improvement (i.e. reduction in bleeding) to complete resolution. Some studies relied on self-reported patient outcomes obtained through questionnaires [16], while others employed more objective, validated scoring systems, such as the Giamundo score, Sodergren hemorrhoidal severity score, HD bleeding grade, Hemorrhoidal Disease Symptom Score, and Short Health Scale for HD [30, 32, 33]. This variability complicates direct comparisons across studies.

Another source of heterogeneity was the follow-up duration, which tended to be shorter in earlier publications and progressively longer in more recent studies. Additionally, some studies reported follow-up as a mean value, rather than at a fixed time point, implying that patient assessments occurred at varying intervals [29, 31, 32]. This inconsistency may have influenced reported outcomes and recurrence rates.

Highly experienced specialists dedicated to coloproctology were all committed to performing all necessary procedures to achieve a tailored approach to HD and had particular proficiency in sclerotherapy. Such expertise may not be common across all clinical settings and could potentially limit the generalizability of our findings. Nevertheless, with appropriate training and adherence to the current standardized protocol, similar outcomes may be readily achievable in other centers.

Further limitations include the heterogeneous study designs, with a mix of retrospective and prospective studies, and the limited number of randomized controlled trials (RCTs) available for high-level evidence. These factors should be considered when interpreting the findings and drawing conclusions regarding the long-term effectiveness and safety of polidocanol foam sclerotherapy. Future research should aim to address these limitations by incorporating standardized definitions of treatment success, longer follow-up periods, and larger multicenter randomized trials to validate these findings.

## Conclusions

The evolution of polidocanol foam sclerotherapy marks a significant advancement in the treatment of HD. This expert survey highlights its potential as a preferred minimally invasive therapy for grade II-III hemorrhoids. Future efforts should focus on refining procedural techniques, optimizing follow-up strategies, and expanding its indications through high-quality evidence.

## Supplementary Information

Below is the link to the electronic supplementary material.

Supplementary file1 (PDF 138 KB)

## Data Availability

The datasets generated and/or analyzed during the current study are available from the corresponding author on reasonable request.
