# Correction: Real-world use of polidocanol foam sclerotherapy for hemorrhoidal disease: insights from an international survey and systematic review with clinical practice recommendations

**DOI:** 10.1007/s13304-025-02337-4

**Published:** 2025-08-04

**Authors:** Gaetano Gallo, Ugo Grossi, Veronica De Simone, Arcangelo Picciariello, Elia Diaco, Pin Fan, Hongbo He, Jun Li, Hongcheng Lin, Marco La Torre, Rita Laforgia, Pierluigi Lobascio, Hui Ma, Francesco Pata, Roberto Perinotti, Vincent De Parades, Mauro Pozzo, Alberto Realis Luc, Paulo Salgueiro, Adam Skowronski, Pingliang Sun, Mario Trompetto, Roberta Tutino, Chen Wang, Zhenyi Wang, Zhenquan Wang, Jiong Wu, Yuru Zhang, Shipeng Zhao, Xiandong Zeng, Vitor Fernandes, Karl-Heinz Moser, Donglin Ren, Pierpaolo Sileri, Gianpiero Gravante

**Affiliations:** 1 Department of Surgery, Sapienza University of Rome, Rome, Italy; 2 Surgery Unit 2, Regional Hospital Treviso ‘Cittadella della Salute’, Piazzale Ospedale 1, 31100 Treviso, Italy; 3 Proctology and Pelvic Floor Surgery Unit, Ospedale Isola Tiberina-Gemelli Isola, 00186 Rome, Italy; 4 Department of Experimental Medicine, University of Salento, Lecce, Italy; 5 Minerva Surgical Service, Catanzaro, Italy; 6 The First Affiliated Hospital of USTC, Anhui Provincial Hospital, Hefei, China; 7 West China Hospital of Sichuan University, Chengdu, China; 8 The Affiliated Hospital of Southwest Medical University, Luzhou, China; 9 The Sixth Affiliated Hospital of Sun Yat-Sen University, Guangzhou, China; 10 Department of Surgery, Ospedale Cristo Re, Rome, Italy; 11 Department of Precision and Regenerative Medicine and Jonic Area (DiMePRe-J), Section of Surgery, General Surgery Unit—Hospital University of Bari, Piazza Giulio Cesare 11, 70124 Bari, SE Italy; 12 The First Affiliated Hospital of Guangxi Medical University, Nanning, China; 13 Department of Pharmacy, Health and Nutritional Sciences, University of Calabria, Cosenza, Italy; 14 General Surgery, SS Colo-Rectal and Proctological Surgery, Biella Hospital, Ponderano, Biella, Italy; 15 Institut Léopold Bellan, Groupe Hospitalier Paris Saint-Joseph, Service de Proctologie Médico-Chirurgicale, Paris, France; 16 Department of Colorectal Surgery, S. Rita Clinic, Vercelli, Italy; 17 Department of Gastroenterology, Hospital de Santo António, Centro Hospitalar Universitário Do Porto, Largo Prof. Abel Salazar, 4099-001 Porto, Portugal; 18 Centrum Medyczne PZU Zdrowie Artimed, Kielce, Poland; 19 Department of General and Emergency Surgery, AOU Città della Salute e della Scienza, Turin, Italy; 20 Longhua Hospital, Shanghai University of Traditional Chinese Medicine, Shanghai, China; 21 Yueyang Hospital of Integrated Traditional Chinese and Western Medicine, Shanghai University of Traditional Chinese Medicine, Shanghai, China; 22 The Second Affiliated Hospital of Hunan University of Traditional Chinese Medicine, Hunan University of Chinese Medicine, Changsha, China; 23 Beijing Rectum Hospital, Beijing, China; 24 Hebei Medical University Third Hospital, Shijiazhuang, Hebei China; 25 the Fourth People’s Hospital of Shenyang, Shenyang, Liaoning China; 26 Gastroenterology Department, Hospital Garcia de Orta, Avenida Torrado da Silva, 2801-951 Almada, Portugal; 27 “Suedstadt” Surgical Group Practise, Karolingerring 31, 50678 Cologne, Germany; 28 Colorectal Surgery Unit, IRCCS San Raffaele Scientific Institute, Vita-Salute University, Via Olgettina 60, 20132 Milan, Italy; 29 Department of General Surgery, Azienda Sanitaria Locale ASL Lecce, Casarano, Italy

**Correction: Updates in Surgery** 10.1007/s13304-025-02258-2

In this article author name Vincent De Parades was incorrectly written as Vincent Parades.

The original article has been corrected.

