# Supplementary material for: Real-world use of polidocanol foam sclerotherapy for hemorrhoidal disease: insights from an international survey and systematic review with clinical practice recommendations
Source: Updates Surg. 2025 Jun 6;77(5):1439–50. doi: 10.1007/s13304-025-02258-2 (PMC12420758; doi:10.1007/s13304-025-02258-2)
Supplement: Supplementary file 1 — Supplementary file1 (PDF 138 KB) [file 13304_2025_2258_MOESM1_ESM.pdf]

Results

Survey 886488

Number of records in this query:	30
Total records in survey:	30
Percentage of total:	100.00%

Summary for Gender

Gender

Answer	Count	Percentage
Female (A1)	5	16.67%
Male (A2)	25	83.33%
No answer	0	0.00%

Summary for Age

Age range (years)

Answer	Count	Percentage
25-30 (A1)	0	0.00%
31-40 (A2)	3	10.00%
41-50 (A3)	13	43.33%
51-60 (A4)	11	36.67%
61-70 (A5)	3	10.00%
71-80 (A6)	0	0.00%
No answer	0	0.00%

Summary for Centre

Centre

Answer	Count	Percentage
Academic (A1)	12	40.00%
Teaching (A2)	12	40.00%
Non teaching (A3)	6	20.00%
No answer	0	0.00%

Summary for Specialty

Specialty

Answer	Count	Percentage
General surgery (A1)	4	13.33%
Colorectal surgery (A2)	16	53.33%
Other	10	33.33%
No answer	0	0.00%

ID	Response
9	gastroenterologist and surgical proctologist
16	gastoenterology
20	Anorectal surgery
22	Anorectal department
23	Angiology
24	Proctology
27	Gastroenterology
35	Anorectal surgery
40	Gastroenterologe
52	Proctology

Summary for Indications

Reasons for sclerotherapy use

**HD Goligher grade I**	**23**	**76.67%**
HD Goligher grade II	27	90.00%
HD Goligher grade III	18	60.00%
HD Goligher grade IV	8	26.67%
Ulcerative colitis	1	3.33%
Crohn's disease	1	3.33%
Pregnancy	4	13.33%
Elderly patients with multiple comorbidities	23	76.67%
Heart disease or patients on anticoagulant therapy or antiplatelets	18	60.00%
Bridge to surgery	13	43.33%
Associated with other office treatments (e.g. banding)	11	36.67%
Associated with surgery for HD (e.g. excisional procedure)	9	30.00%
Other	1	3.33%

**ID**	**Response**
2	Efficacy demonstrated, Cost-effective, repeatable
9	Best indication: bleedings
10	good for bleeding
14	Sclerotherapy can effectively alleviate the symptoms of hemorrhoids such as bleeding and protrusion, and it can also reduce the size of hemorrhoid nuclei.
27	Minimally invasive procedure with demonstrated safety and efficacy in this group of patients
30	[illegible]
31	Bleeding persistent
32	bleeding
37	With bleeding
43	Liquid
46	In case of bleeding non responsive to medical treatment
49	Sclerotherapy can reduce the size of hemorrhoids and effectively alleviate the symptoms of bleeding and protrusion caused by hemorrhoidal disease.
52	Bleeding
2	Efficacy demonstrated, Cost-effective, repeatable
9	Best indication: bleedings and little prolapse
10	good for bleeding and mild peolapse
14	Sclerotherapy can effectively alleviate the symptoms of hemorrhoids such as bleeding and protrusion, and it can also reduce the size of hemorrhoid nuclei.
27	Minimally invasive procedure with demonstrated safety and efficacy in this group of patients
30	[illegible]
31	Bleeding persistent
32	bleeding
37	with bleeding or/and prolapse
43	Foam
46	In case of bleeding non responsive to medical treatment
49	Sclerotherapy can reduce the size of hemorrhoids and effectively alleviate the symptoms of bleeding and protrusion caused by hemorrhoidal disease.
52	Bleeding, prolapse
2	Potentially effective (resolution Vs symptoms control)
14	Sclerotherapy can effectively alleviate the symptoms of hemorrhoids such as bleeding and protrusion, and it can also reduce the size of hemorrhoid nuclei.
27	Minimally invasive procedure with demonstrated safety and efficacy in grade III selected patients
30	[illegible]
31	Bleeding persistent in unfit for other treatment
32	bleeding or bridge to surgery
37	with bleeding or/and prolapse
49	Sclerotherapy can reduce the size of hemorrhoids and effectively alleviate the symptoms of bleeding and protrusion caused by hemorrhoidal disease.
52	Symtomatic with contraindications for LHP OR DGHAL

2	As bridge to surgery or in frail bleeding patients with a high risk of complications in case of surgery
14	Sclerotherapy can effectively alleviate the symptoms of hemorrhoids such as bleeding and protrusion, and it can also reduce the size of hemorrhoid nuclei.
31	Bleeding persistent in unfit for m-m
37	After hemorrhoidectomy
49	Sclerotherapy can reduce the size of hemorrhoids and effectively alleviate the symptoms of bleeding and protrusion caused by hemorrhoidal disease.
10	safer than other treatment
14	Sclerotherapy can help alleviate symptoms for pregnant women with hemorrhoids, and it is generally considered safe.
49	Sclerotherapy is safe, effective, minimally invasive, and causes little pain.
2	As bridge to surgery or in frail bleeding patients with a high risk of complications in case of surgery
10	safer than other treatment
20	Security
27	Minimally invasive procedure with no apparent drug interactions
30	[illegible]
32	operative risk
37	With bleeding
38	[illegible]
43	Blood thinner
46	To avoid surgery
2	Effective with the chance to control symptoms without the risk of suspension (rebound hyper coagulability) or postoperative bleeding
10	safer than other treatment
20	Security
27	Minimally invasive procedure with demonstrated safety and efficacy in this group of patients; no need to stop antithrombotics
30	[illegible]
38	[illegible]
46	To avoid surgery
2	Potential downstage of the disease to consent mini-invasiva treatment. Or useful to postpone surgery in exceptional circumstances (e.g. Pandemic)
10	more effective for bleeding symptoms
14	Sclerotherapy can serve as a bridge between conservative treatment and surgery.Patients who do not respond to sclerotherapy may consider surgery.
27	Minimally invasive procedure with demonstrated efficacy in this group of patients
32	when the waiting list requires months
49	Sclerotherapy can reduce the size of hemorrhoids and effectively alleviate the symptoms of bleeding and protrusion caused by hemorrhoidal disease.
2	Sclerobanding may theoretically improve both techniques with a synergic effect. Safety profile and feasibility already demonstrated in two pilot studies in literature
20	Preventing postoperative massive bleeding
30	[illegible]
35	Prevention of bleeding during sloughing of the hemorrhoids.
49	After surgically removing larger hemorrhoids, smaller hemorrhoids can be treated with sclerotherapy.
52	Rarely
14	Large hemorrhoid nuclei can be surgically removed, while smaller ones can be treated with sclerotherapy.
27	I use it often, after surgery, when there is remaining hemorrhoidal pedicles
49	After surgically removing larger hemorrhoids, smaller hemorrhoids can be treated with sclerotherapy.
27	Apparently safe in patients with hemorrhagic dyscrasia

Summary for Contraindications

Are there instances where you absolutely contraindicate sclerotherapy?

**Active fissure**	**13**	**43.33%**
Anal stenosis	17	56.67%
Anal abscess	22	73.33%
Special conditions (please specify)	9	30.00%
Other	0	0.00%

ID	Response
27	I always try to treat the fissure first
32	infection risk
46	I first treat the fissure
49	The use of sclerotherapy for active fissure may increase the risk of complications.
27	Contraindicated only if it is not possible to perform anoscopy
32	technical difficulties
35	Sclerotherapy will aggravate stenosis.
49	Due to the lack of adequate visualization and operating space, sclerotherapy is not recommended for anal stenosis.
14	Sclerotherapy injections may increase the risk of anal infections, and it should be performed only after the infection has been eliminated.
27	I always try to treat the suppurative disease first
30	[illegible]
32	infection risk
46	Must treat the abscess first
49	Using sclerotherapy for anal abscess has the risk of causing infection spread and is not recommended.
9	anticoagulant therapy
16	perianal Crohn disease, infeccion, pain
27	Active perineal or rectal Crohn's disease/ulcerative colitis
29	pregnancy
37	Bleeding too much but inoperable
38	[illegible]
40	Anal Crohn, infection, pain
47	acute inflammation or diseases of the anal region - chronic anorectal inflammatory diseases
51	Bleeding or prolapse

Summary for PreopRecomm

Preoperative therapy recommendations

**None**	**11**	**36.67%**
Stool softeners	15	50.00%
High-fiber diet	12	40.00%
Increased water intake	9	30.00%
Topical flavonoids	4	13.33%
Systemic flavonoids	11	36.67%
Topical corticosteroids	0	0.00%
Systemic corticosteroids	1	3.33%
Other	0	0.00%

**ID**	**Response**
2	Only in case of chronic constipation or usual hard stool evacuation
14	Softening stool can avoid the spasms of anal sphincter muscles and the increase in local venous pressure caused by forced bowel movements.
27	To reduce defecation straining
32	always
35	Reduce defecation discomfort
38	[illegible]
49	Adjusting diet and developing good bowel habits are beneficial for any type of hemorrhoidal disease.
14	It can help to form a good defecation habits.
27	To reduce defecation straining
35	Reduce defecation discomfort
49	Adjusting diet and developing good bowel habits are beneficial for any type of hemorrhoidal disease.
14	It can help to form a good defecation habits.
27	To reduce defecation straining
32	always
38	[illegible]
49	Adjusting diet and developing good bowel habits are beneficial for any type of hemorrhoidal disease.
27	Drugs apparently effective in the symptomatic treatment of hemorrhoidal disease; prior treatment helps to understand whether there is really an indication for sclerotherapy
2	Only in case of severe symptoms
14	It can effectively reduce the symptoms of hemorrhoid edema and bleeding.
23	Ffpm
27	Drugs apparently effective in the symptomatic treatment of hemorrhoidal disease; prior treatment helps to understand whether there is really an indication for sclerotherapy
32	always
49	Flavonoid medications can effectively improve the symptoms of bleeding and edema associated with hemorrhoidal disease.
49	Corticosteroids can be effective in reducing inflammatory reactions and relieving pain.

Summary for DurationPreopTherapy

Duration of preoperative therapy

Answer	Count	Percentage
(A2)	9	47.37%
2 weeks (A3)	3	15.79%
4 weeks (A4)	6	31.58%
6 weeks (A5)	1	5.26%
8 weeks (A6)	0	0.00%
>8 weeks (A7)	0	0.00%
Other	0	0.00%
No answer	0	0.00%

ID	Response

Summary for Preparation

Patients preparation

Answer	Count	Percentage
Single enema a few hours before the procedure (SQ001)	17	56.67%
Enemas the night before and a few hours before the procedure (SQ002)	5	16.67%
None (SQ003)	10	33.33%
Other	1	3.33%

ID	Response
40	Usual fisiológico defecation before

Summary for Position

What's your preferred and most used patient's position for sclerotherapy?

Answer	Count	Percentage
Sims - left lateral (A1)	16	53.33%
Lloyd Davies / lithotomy (A2)	7	23.33%
Jack-knife - prone (A3)	5	16.67%
Other	2	6.67%
No answer	0	0.00%

ID	Response
16	left lateral position wih flexion of legs

Summary for Setting

Main setting for sclerotherapy

Answer	Count	Percentage
Office (A1)	16	53.33%
Day surgery (A2)	9	30.00%
Inpatient (A3)	5	16.67%
Other	0	0.00%
No answer	0	0.00%

ID	Response

Summary for Percentage

Percentage of polidocanol used

Answer	Count	Percentage
3% (A1)	17	65.38%
2% (A2)	2	7.69%
1% (A3)	7	26.92%
Other	0	0.00%
No answer	0	0.00%

ID	Response

Summary for Type

Sclerotherapy method

Answer	Count	Percentage
Transanal only (A1)	24	80.00%
Endoscopic only (A2)	0	0.00%
Both methods (A3)	6	20.00%
No answer	0	0.00%

Summary for Anoscope

Transanal with anoscope?

Answer	Count	Percentage
Yes (A1)	30	100.00%
No (A2)	0	0.00%
No answer	0	0.00%

Summary for FoamFormation

Foam formation method in clinical practice

Answer	Count	Percentage
Tessari (A1)	12	46.15%
Automated device (e.g., Varixio) (A2)	2	7.69%
EasyFoam Kit (A3)	10	38.46%
Variable (A4)	0	0.00%
Other	2	7.69%
No answer	0	0.00%

ID	Response
16	3-way tap and 2 syringe 20 cc
40	3 way tap and 2 seringues immediately before inj

Summary for CaliberNeedle

Gauge of needle used

Answer	Count	Percentage
17 G (A2)	2	6.67%
18 G (A3)	2	6.67%
19 G (A4)	0	0.00%
20 G (A5)	7	23.33%
21 G (A6)	1	3.33%
22 G (A7)	5	16.67%
23 G (A8)	4	13.33%
24 G (A9)	3	10.00%
25 G (A10)	3	10.00%
26 G (A11)	0	0.00%
Other	3	10.00%
No answer	0	0.00%

ID	Response
16	green iv
22	1ml
40	Iv green

Summary for Anesthesia

Type of anesthesia used

Answer	Count	Percentage
None (A1)	15	50.00%
Topical anesthetic (e.g. lidocaine gel) (A2)	9	30.00%
Injection of local anesthetic (A3)	1	3.33%
Sedation (A4)	0	0.00%
Spinal anesthesia (A5)	5	16.67%
Other	0	0.00%
No answer	0	0.00%

ID	Response

Summary for InjectionTarget

Injection target of sclerosing agent

Answer	Count	Percentage
Mucosal/submucosal (A2)	10	33.33%
Intra-hemorrhoidal (A3)	19	63.33%
Other	1	3.33%
No answer	0	0.00%

ID	Response
52	Both

Summary for VolumeAgent

Volume of sclerosing agent administered per pile (NB for foam, refer to total volume air+agent injected)

Answer	Count	Percentage
2 ml (A2)	20	66.67%
3 ml (A3)	5	16.67%
4 ml (A4)	1	3.33%
5-10 ml (A5)	4	13.33%
Comments	8	26.67%
No answer	0	0.00%

ID	Response
8	1 ml anterior quadrant
9	Limited quantity
10	above the pile 2-3ml<br>pile 2-3ml
14	range of 2-3 ml
16	Depending of plasticity of hemorroidal cushion - in between 10 and 30 ml
27	The volume depends on the resistance felt during the instillation of the sclerosant
32	for anterior piles 1/1.5 ml
52	Always foam, sometimes less then 2ml/pile

Summary for TotalVolume

Maximum volume of sclerosing agent administered per treatment

Answer	Count	Percentage
6 ml (A2)	8	26.67%
8 ml (A3)	3	10.00%
10 ml (A4)	10	33.33%
12 ml (A5)	4	13.33%
14 ml (A6)	1	3.33%
16 ml (A7)	0	0.00%
18 ml (A8)	0	0.00%
20+ ml (A9)	4	13.33%
Comments	3	10.00%
No answer	0	0.00%

ID	Response
10	usually less than10ml when accompany with other HD treatment
14	range of 10-15 ml
16	40 ml

Summary for Adjustements

Adjustment of agent volume based on hemorrhoidal disease grade

Answer	Count	Percentage
Yes (A1)	25	83.33%
No, I always administer a standard quantity (A2)	5	16.67%
Comments	3	10.00%
No answer	0	0.00%

ID	Response
14	The dosage of sclerosing agent is determined based on the size and severity of the hemorrhoid.
27	The volume depends on the resistance felt during the instillation of the sclerosant; Often more advanced degrees require larger volumes
49	For larger hemorrhoids, the injection dose can be appropriately increased during sclerotherapy.

Summary for NumberOfPiles

Number of piles treated per session

Answer	Count	Percentage
1 (A1)	1	3.33%
2 (A2)	1	3.33%
3 (A3)	16	53.33%
>3 if other bleeding areas evident (A4)	12	40.00%
Comments	4	13.33%
No answer	0	0.00%

ID	Response
14	Too many injection sites may lead to complications such as anal stenosis.
27	Normally 1 or 2 pyles since we believe that, when injecting large volumes, the sclerosant disperses throughout the hemorrhoidal territory
32	it depends on the clinical scenario
49	Single injection treatment should not exceed 3 piles□ and the total dosage should not exceed the recommended dose stated in the instructions.

Summary for PostopRecomm

Postoperative therapy recommendations

None	2	6.67%
Stool softeners	23	76.67%
High-fiber diet	16	53.33%
Increased water intake	20	66.67%
Topical flavonoids	1	3.33%
Systemic flavonoids	17	56.67%
Topical corticosteroids	0	0.00%
Systemic corticosteroids	2	6.67%
Other	3	10.00%

ID	Response
40	Laxative if need, paracetamol for pain if need, hemorrhoids ointment without corticoids
10	lactulose
14	It helps to cultivate a good defecation habit.
27	Whenever necessary; with the aim of reducing defecatory straining
32	always
35	Adjusting bowel movements to accelerate recovery
43	Mucofalk
49	Adjusting diet and developing good bowel habits are beneficial for any type of hemorrhoidal disease.
14	It helps to cultivate a good defecation habit.
27	Whenever necessary; with the aim of reducing defecatory straining
35	Adjusting bowel movements to accelerate recovery
49	Adjusting diet and developing good bowel habits are beneficial for any type of hemorrhoidal disease.
14	It helps to cultivate a good defecation habit.
27	Whenever necessary; with the aim of reducing defecatory straining
35	Adjusting bowel movements to accelerate recovery
38	[illegible]
49	Adjusting diet and developing good bowel habits are beneficial for any type of hemorrhoidal disease.
27	I use it per protocol, although recognizing that there is no scientific evidence for its use
2	Diosmine 500 mg twice a day X 15 days, then once a day for the following 15 days
14	It can effectively reduce the symptoms of hemorrhoid edema and bleeding.
27	I use it per protocol, although recognizing that there is no scientific evidence for its use
32	always
49	Flavonoid medications can effectively improve the symptoms of bleeding and edema associated with hemorrhoidal disease.
49	Corticosteroids can be effective in reducing inflammatory reactions and relieving pain.
9	paracetamol
16	occasionally antibiotic
40	Paracetamol for pain and topic hemorrhoids ointment without corticoid

Summary for DurationPostopTherap

Duration of postoperative therapy

Answer	Count	Percentage
(A2)	10	35.71%
2 weeks (A3)	9	32.14%
4 weeks (A4)	8	28.57%
6 weeks (A5)	1	3.57%
8 weeks (A6)	0	0.00%
>8 weeks (A7)	0	0.00%
Other	0	0.00%
No answer	0	0.00%

ID	Response

Summary for SecondSession

Do you routinely schedule a second sclerotherapy session?

Answer	Count	Percentage
Yes (A1)	7	23.33%
No (A2)	23	76.67%
No answer	0	0.00%

Summary for CriteriaSecond

Criteria indicating the need for a second session

Answer	Count	Percentage
Not applicable as I never do a second session (A1)	2	6.67%
Symptoms or Patient-reported outcome measures (PROMs) (A2)	23	76.67%
Patient's clinical diary (A3)	5	16.67%
Comments	3	10.00%
No answer	0	0.00%

ID	Response
14	Patients who experience recurrence after the first sclerotherapy treatment can consider undergoing sclerotherapy again.
27	I usually schedule a reassessment in 1 to 2 months; if the patient maintains hemorrhoidal symptoms and anoscopy reveals significant hemorrhoidal disease, a second treatment session is performed
49	If sclerotherapy is effective and the patient requests, a second treatment can be scheduled.

Summary for Timeframe

Minimum interval from first to second sclerotherapy treatment (if necessary)

Answer	Count	Percentage
1 week (A2)	1	3.33%
2 weeks (A3)	3	10.00%
3 weeks (A4)	1	3.33%
4 weeks (A5)	15	50.00%
5 weeks (A6)	1	3.33%
6 weeks (A7)	4	13.33%
7 weeks (A8)	0	0.00%
8 weeks (A9)	4	13.33%
Other	1	3.33%
No answer	0	0.00%

ID	Response
22	12 weeks

Summary for Efficacy

Efficacy assessment methods

PROMs (include scores used: HDSS-SHS-Giamundo-Sodergreen-Vaizey-others)	12	40.00%
Anoscopy	20	66.67%
Hemoglobin levels	6	20.00%
Patient's subjective experience	24	80.00%
Other	0	0.00%

ID	Response
14	This method is more suitable for patients with hemorrhoids.
27	HDSS
32	to be administered to all patients
49	The treatment effect should be comprehensively evaluated from several aspects, including symptom improvement, patient experience, and objective examination.
16	Only if the patient keeps blood and prolapse
27	I only apply treatment if the hemorrhoidal pyles allow it (sometimes the fibrosis resulting from previous treatments reduces vascular compliance; I always use intra-hemorrhoidal injection)
32	performed during follow-up
38	[illegible]
49	The treatment effect should be comprehensively evaluated from several aspects, including symptom improvement, patient experience, and objective examination.
2	In case of severe anemia
38	[illegible]
27	I only treat patients who are symptomatic, even if significant hemorrhoidal disease is identified during anoscopy
32	diary
38	[illegible]
49	The treatment effect should be comprehensively evaluated from several aspects, including symptom improvement, patient experience, and objective examination.
52	Presisting / recurrent symtomps

Summary for FupTiming

Minimum follow-up timing after sclerotherapy session

Answer	Count	Percentage
1 day (A2)	3	10.00%
2 days (A3)	0	0.00%
3 days (A4)	1	3.33%
5 days (A5)	0	0.00%
7 days (A6)	10	33.33%
15 days (A7)	5	16.67%
30 days (A8)	10	33.33%
Other	1	3.33%
No answer	0	0.00%

ID	Response
52	Usually 8 weeks, unless any unusual symptoms like pain, fever, severe bleeding - in thatbcases ASAP.

Summary for FupMethods

Follow-up methodology

Answer	Count	Percentage
Remote (A1)	4	13.33%
In-person (A2)	26	86.67%
Other	0	0.00%
No answer	0	0.00%

ID	Response
